# Multiple therapeutic effect of endothelial progenitor cell regulated by drugs in diabetes and diabetes related disorder

**DOI:** 10.1186/s12967-017-1280-y

**Published:** 2017-08-31

**Authors:** Rashmi K. Ambasta, Harleen Kohli, Pravir Kumar

**Affiliations:** Molecular Neuroscience and Functional Genomics Laboratory, Department of Biotechnology, DTU, Delhi, India

**Keywords:** Diabetes, EPC, eNOS, Metformin, ROS, NOX

## Abstract

**Background:**

Reduced levels of endothelial progenitor cells (EPCs) counts have been reported in diabetic mellitus (DM) patients and other diabetes-related disorder. EPCs are a circulating, bone marrow-derived cell population that appears to participate in vasculogenesis, angiogenesis and damage repair. These EPC may revert the damage caused in diabetic condition. We aim to identify several existing drugs and signaling molecule, which could alleviate or improve the diabetes condition via mobilizing and increasing EPC number as well as function.

**Main body:**

Accumulated evidence suggests that dysregulation of EPC phenotype and function may be attributed to several signaling molecules and cytokines in DM patients. Hyperglycemia alone, through the overproduction of reactive oxygen species (ROS) via eNOS and NOX, can induce changes in gene expression and cellular behavior in diabetes. Furthermore, reports suggest that EPC telomere shortening via increased oxidative DNA damage may play an important role in the pathogenesis of coronary artery disease in diabetic patients. In this review, different type of EPC derived from different sources has been discussed along with cell-surface marker. The reduced number and immobilized EPC in diabetic condition have been mobilized for the therapeutic purpose via use of existing, and novel drugs have been discussed. Hence, evidence list of all types of drugs that have been reported to target the same pathway which affect EPC number and function in diabetes has been reviewed. Additionally, we highlight that proteins are critical in diabetes via polymorphism and inhibitor studies. Ultimately, a lucid pictorial explanation of diabetic and normal patient signaling pathways of the collected data have been presented in order to understand the complex signaling mystery underlying in the diseased and normal condition.

**Conclusion:**

Finally, we conclude on eNOS-metformin-HSp90 signaling and its remedial effect for controlling the EPC to improve the diabetic condition for delaying diabetes-related complication. Altogether, the review gives a holistic overview about the elaborate therapeutic effect of EPC regulated by novel and existing drugs in diabetes and diabetes-related disorder.

## Background

Diabetes is associated with endothelial cell dysfunction and impaired neovascularization and repair mechanism of the body. Endothelial Progenitor Cells are those cells that contribute to vascularization and angiogenesis during the embryo development and adult stage. When these cells were isolated by Asahara et al. in 1997 from the peripheral blood circulation [1, 2], an emerging development is made on the study of blood vessel formation and vascular diseases relating to it. EPCs differentiate into mature endothelial cells and lines the lumina of blood vessel wall forming a monolayer called the endothelium which has important functions like maintaining the vascular tone, preventing adhesion of leukocyte and preventing proliferation of underlying smooth muscle cells, anti-inflammatory property and maintaining homeostasis [3]. The endothelial progenitor cells have been isolated from bone marrow [4–6] peripheral blood circulation [7], human umbilical vein [8, 9], human umbilical cord blood [10, 11] etc., by density centrifugation method.

EPCs were a challenge because of the isolation of pure population from the heterogeneous cell population. Many cell-surface markers have been used for identification of EPCs. It is also believed that the EPCs arise from hemangioblast, a precursor to endothelial and hematopoietic stem cells [12, 13]. As the EPCs grow in culture, few surface markers are lost indicating mature population of EPCs. Loss of CD133 (AC133) which is a specific surface marker for the hematopoietic cells from the seeding population is an indication that the cells are differentiating into an endothelial lineage [14] and takes up VEGFR-2, one of the cell-surface markers of EPCs [15] Abb. Commonly used markers used for identification of EPCs are AC133 (CD133), CD34, platelet/endothelial cell adhesion molecule-1 (PECAM-1 or CD31), ^*^Flk-1/KDR (^*^Flk-1 is also known as VEGFR-2 in mouse and KDR is human homolog of VEGFR-2) [1], von Willebrand factor (vWF), vascular endothelial cadherin (VE-Cadherin) [16], endothelial nitric oxide synthase (eNOS) [17], uptake of DiI AcLDL [18, 19] and BS-1 or UEA-1 lectin binding. However, wide range of cell-surface markers shares with other cell types like hematopoietic stem cells (CD133 and CD34), platelets (CD31), megakaryocytes (vWF) and CD14 (monocytes) [20]. The identification of EPC is very important in the therapeutic effect of the same. Hence different type of EPC and its identification have been discussed below.

## Identification of different type of EPC

There are two types of EPC based on the time in culture, and those are early EPC and late EPC as shown in Table 1. Early EPC is elongated, and spindle shaped while late EPC is cobblestone shaped. Early EPC appearance in culture is within 4–7 days while late EPC is 2–4 week in culture. Late EPC is more homogenous compared to the heterogeneous population of early EPC. The life span of late EPC is up to 12 week as compared to the short life span of an early EPC i.e. 3–4 week. The proliferation potential of late EPC is higher compared to the low proliferation potential of early EPC. Late EPC has a strong expression of VE-cadherin, Flt-1, KDR, e-NOS, vWF while early EPC has a weak expression level of VE cadherin and KDR. Late EPC has high competency to produce NO compared to early EPC. Late EPC has enhanced neovasculogenesis property compared to only cytokine release property of early EPC. Hence it can be summarized that late EPC should be more preferred for transplantation as it better mimics the EPC property.

**Table 1:** Properties of two different type of EPCs

Characteristics	Early EPCS	Late EPCS
Morphology	Elongated and spindle shape	Cobblestone appearance
Appearance in culture	4–7 days of culture	2–4 weeks
Purity	Heterogeneous group of cells that differentiate from hemangioblasts to mature endothelial cells	Homogeneous and well differentiated. Derived mainly from “early” EPC
Life span	3–4 weeks	Up to 12 weeks
Proliferation potential	Low compared to “late” EPCs	Highly proliferating
Gene expression	Week expression of VE-cadherin and KDR. The level of Flt-1 expression elevated	Strong expression of VE-cadherin, Flt-1, KDR, and e-NOS, vWF
eNOS expression	Less competent endothelial function producing nitric oxide	High competent endothelial function producing nitric oxide

The different types of EPC based on its source of origin are: EPC from peripheral blood, bone marrow derived EPC; embryo derived EPC; cord blood and HUVEC derived EPC. The markers expressed by these EPC are different as shown in Table 2.

**Table 2:** EPC marker

Type of EPC	Marker	References
Peripheral blood circulating EPC	vWF, CD31, CD34, VEGF-R2, CD105, CD146	Untergasser et al. [21]
Peripheral blood circulating EPC	CD34+/CD133+/VEGF-R2+	Capiod et al. [22]
Peripheral blood circulating EPC	CD133−, CD34+, VEGFR-2, VE-cadherin, eNOS, vWF	Hristov, Weber [23]
Peripheral blood circulating EPC	Positive for acetylated LDL, vWF, P1H12, thrombomodulin, flk-1, VE-cadherin, PECAM-1, CD34, CD14-	Lin et al. [24]
Peripheral blood circulating EPC	CD31, Tie-2, and Flk-1, VEGFR-2	Ito et al. [25]; Hill et al. [66]
Bone marrow derived EPC	CD31(+), c-Kit(+), Sca-1(+), Lin(−)	Khoo et al. [26]
Bone marrow derived EPC	VEGFR-2, CD31, VE-cadherin, vWF	Hristov et al. [27]
Bone marrow derived EPC	Flk1(+)CD31(−)CD34(−)	Guo et al. [28]
Bone marrow derived EPC	KDR, vWF, eNOS, VE-cadherin, CD146, uptake of DiI-acetylated LDL and binding of lectin	Yoon et al. [29]
Bone marrow derived EPC	Flk-1, Tie-2, Sca-1, and CD34	Asahara et al. [56]
Embryo derived EPC	Early: Flk-1, PECAM, and tie-2, VEGF; mature: tie-I, MECA-32 antigen, VE-cadherin, and MEC-14.7	Vittet et al. [30]
Embryo derived EPC	BS Lectin, vWF, DiI Ac-LDL	Doetschman et al. [31]
Embryo derived EPC	CD31, CD34, Flk-1, VE-cadherin, and vWF, uptake of DiI-ac-LDL	Li et al. [32]
Embryo derived EPC	eNOS, VEGF Flk-1 and Flt-1, VE-cadherin, CD34, PECAM-1	McCloskey et al. [33]; Glaser et al. [34]
Cord blood EPC	UEA Lectin Binding, VEGF receptor-2, vWF, CD31, CD34, eNOS, Ac-LDL-uptake	Schmidt et al. [35]
Cord blood EPC	DiI ac-LDL, KDR, VE-cadherin, CD31, vWF, CD45−	Murohara et al. [36]
Cord blood EPC	VEGFR-2, KDR, VE Cadherin (CD144), CD18, and CD61, acetylated LDL uptake and ulex lectin binding	Ahrens et al. [37]
Cord blood EPC	Positive: CD31, VE-cadherin, and vWF Negative: CD45, CD90, α-SMA	Lin et al. [38]
Cord blood EPC	Flt-1/VEGFR-1, ecNOS, VE-cadherin, von Willebrand factor, and secreted VEGF	Jang et al. [39]
HUVEC derived EPC	vWF, CD31, CD34, UEA-1 lectin	Hughes et al. [40]
HUVEC derived EPC	CD133, P1H12, VEGFR2, PECAM, and endoglin, ICAM1	Bagley et al. [41]
HUVEC derived EPC	Ac LDL, VEGFR1, VEGFR2, FLT-1 and KDR	Carvalho et al. [42]

The peripheral blood derived EPC markers are CD31, Tie-2, and Flk-1, VEGFR-2 while bone marrow derived EPC markers are VEGFR-2, CD31, VE-cadherin, vWF. The embryo derived EPC markers are eNOS, VEGF Flk-1 and Flt-1, VE-cadherin, CD34, PECAM-1. The cord blood derived EPC markers are DiI ac-LDL, KDR, VE-cadherin, CD31, vWF, CD45- while the HUVEC derived EPC markers are CD133, P1H12, VEGFR2, PECAM, and endoglin, ICAM1. Hence we can summarize that the common markers for EPC derived from several sources can be CD31, VEGF R1/2, Flk1, VE-cadherin, vWF, eNOS, CD34, CD133 as shown in Table 2.

There are at least two different types of EPCs that have been identified from the peripheral blood circulation [43] like early and late EPC [44]. EPCs in circulation and endothelial cells on the vessel wall are always in contact with the blood flow which exerts a laminar shear stress. Experiments conducted shows that EPCs are elongated to the direction of flow and also seem to induce proliferation and tube formation [45, 46]. Flk-1^+^ embryonic stem cells also have reported to differentiate into vascular endothelial cells under fluid shear stress [47]. Flow dynamics have also shown to mediate the phosphorylation of Akt suppressing apoptosis [48] Also the mechanical stimulation (fluid shear stress) helps in the signal transduction cascade of PECAM-1 in activating eNOS and Akt [49].

EPCs are located in the microenvironment of the bone marrow and get into the circulation due to different cytokines and signals from the activation. Endothelial nitric oxide synthase [50], granulocyte colony stimulating factor (G-CSF) or granulocyte macrophage colony stimulating factor (GM-CSF) has shown to induce mobilization of the bone marrow cells into the circulation and helps in proliferation of EPCs [51–53]. Vascular damage or tumor releases various growth factors and cytokines, which help to mobilize the progenitor stem cells from the bone marrow to the site of injury [54, 27]. VEGF is a potential factor having angiogenic property and considered to be one of the major stimulants for the mobilization of the EPCs via Akt pathway by the phosphorylation of eNOS at Ser-1177 [55] resulting in the increase in the number of EPCs and capillary formation [56, 57].

This is believed to occur by the following: matrix metalloproteinase-9 activation leading to the transformation of membrane-bound kit ligand to soluble kit ligand and movement of cKit-positive hemangioblast cells [58]. Migration towards various cytokines, growth factors, including VEGF, FGF, etc., released from the damaged vessel or tumour [56].

Culture of CD34^+^ cells yielded a phenotype of endothelial cell [59] and when cultured together with CD34^−^ cells contributed to neovascularization [60]. Increase in CCN1, a cysteine-rich heparin binding protein has shown to induce the release of cytokines and MMP-9 in CD34^+^ cells resulting in the mobilization and proliferation towards endothelial cells [61]. Mesenchymal stem cells which are purely negative for endothelial cells could differentiate into endothelial lineage when confluent MSCs were incubated with 2% FCS and VEGF [62]. Similarly AC133^+^ cells were also able to differentiate into endothelial cells with the additional FBS and horse serum contained with VEGF.

EPCs release important factors like Nitric Oxide (NO), endothelin-1, angiotensin-converting enzyme (ACE) [63]. Nitric oxide (NO) was found to be a vasorelaxing agent released due to the conversion of l-arginine to l-citrulline in the presence of NADPH catalyzed by endothelial nitric oxide synthase (eNOS). Furthermore, it prevents leukocyte and platelet adhesion and proliferation of smooth muscle cells. Endothelin-1, acts as the vasoconstrictor by stimulating ET_A_ receptors in vascular smooth muscle and acts as vasodilator stimulating ET_B_ receptors in endothelial cells. Angiotensin-converting enzyme (ACE) is a vasoconstrictor agent and Ang-II is released due to the conversion of Ang-I to Ang-II by ACE. It also inhibits bradykinin (vasodilator). The markers of EPC are very clearly derived from different source but whether the EPCs are functional or non-functional in the diabetes condition is discussed below via the mentioned mechanism.

## Functional properties of EPC

Diabetes mellitus severely affects the circulating EPC number and function which also affect the repair mechanism in the same patients. EPCs can be mobilized from bone marrow (BM) into peripheral circulation at sites of injury. Studies report that circulating EPC is reduced in terms of decreased proliferation, adhesion and vasculogenesis in DM patients. Additionally, in vitro hyperglycemia or a diabetic intrauterine environment has shown diminished EPC colony formation suggesting that reduced EPC number and function is directly correlated to diabetic condition in the body. Moreover, decreased NO and VEGF have also been reported in diabetic condition. Amongst the different signaling mechanism reported, eNOS dysfunction and altered cytokine gradient like SDF 1, VEGF, CXCR4 plays major role in impairment of EPC mobilization. Homing of circulating EPC to sites of injury can contribute to vascular repair. It has been shown that blockage of SDF 1 or CXCR4 can prevent the recruitment of EPC to injured sites indicating that indeed these cytokines are critical in recruiting EPC to sites of injury. The other signaling pathway affecting EPC is partially mediated through Ras/ERK/VEGF and PI3K/Akt/eNOS regulation along with the other cytokines. Recently, reports have indicated that altered expression of microRNA 126 and 130a also has been implicated in EPC dysfunction through VEGF/PI3K/Akt/eNOS.

Clinical studies report that statins increases the number and function of circulating EPC by increasing NO and reducing oxidative stress and apoptosis of EPCs. Blocking of the renin angiotensin system with ACE inhibitors has been shown to increase EPC number in DM patients possibly by anti-inflammatory and antioxidative action. Recently, it has been proven that a combination of statins as well as ACE blockers can produce the synergistic effects in increasing the EPC number/function in diabetic patients. Alternatively, lifestyle modification like exercise and weight loss can also exhibit the same effect in EPC number and function. Direct administration of cytokines SDF1, VEGF or a cocktail of cytokines can also reverse EPC dysfunction in DM patients. Additionally, microRNA based treatment might also reverse the dysfunction EPC in DM.

Hence it is crystal clear that in DM patients’ number and function of EPC is affected via VEGF/PI3K/Akt/eNOS pathway along with cytokines SDF1, CXCR4 which plays a critical role for recruitment of EPC to damaged sites which in turn affects the repair mechanism in these patients.

## Impact of EPC circulation and function on clinical parameter and prognosis of DM patients

The burden of diabetes mellitus is associated with other cardiovascular complications in the body which arises due to low number and function of circulating EPC. The elevated level of circulating EPC contributes to the repair mechanism induced by PI3K/Akt/eNOS pathway via HIF1 alpha and IL-8 expression. Hence the number of circulating EPC represents biomarker of global complication burden in diabetic. The damage caused to the body after prolonged hyperglycemia is retinopathy, limb ischemia, neuropathy and other cardio and microvascular complication. The higher the damage level, the higher should be the circulating EPC to repair the damage. However, in case of DM, the number and function of circulating EPC is low. Interestingly, it has been shown that via multifactorial intervention of well-known drugs like metformin, statins, ACE blockers the number and function of circulating EPC can be improved in diabetic patients to repair the damage of micro and macroangiopathy. The high level of circulating EPC also improves the clinical parameter in diabetes like blood-glucose level, insulin level and organ repair like pancreas islet and kidney repair. It has been demonstrated that cotransplantation of EPCs and pancreatic islet ensures long lasting normoglycemic condition. EPC mobilization improves the ischemia induced neovascularization, diabetic wounds, ischemia stroke model in diabetic patients.

## Pathogenesis of diabetes induced by impaired EPC function

Impaired EPC in diabetic patients leads to diabetic retinopathy, impaired neovascularization and several other complications in DM patients.

Circulating number of EPCs help to predict the future cardiovascular events [64] and are found to be decreased in the patients with diabetes, cardiovascular risks [65, 66], peripheral vascular complications [67] chronic renal failure [68], hypercholesterolemia [69]. Senescence of endothelial cells due to aging, impaired migration from the bone marrow and molecular mechanisms like increased activity of caspase-3 causing apoptosis in EPCs, reduced expression of telomerase repeating factor-2 (TRF-2) inhibiting the migration of EPCs [70], modify the differentiation potential of bone marrow cells to EPCs under hyperglycemic condition [23, 71, 72] are some of the causes in reduced EPCs number.

Response to inflammatory cytokines like thrombin leads to ‘activation’ of endothelial cells causing endothelial ‘dysfunction’. This disrupts the VE-Cadherin activity in the endothelial cells causing them to lose the anti-permeability property of the endothelium and forming gaps between the endothelial cells allowing monocytes and leukocytes to penetrate into them. The cause of this property might be due to the modulation in phosphorylation of proteins and actin-myosin contraction [73, 74]. Nitric oxide synthase produces cardioprotective cytokines including eNOS and inducible NOS (iNOS) [75] and regulates the hypertension [76]. Under ischemia where O_2_ are deprived (hypoxia); VEGF gene is upregulated via phosphatidylinositol 3-kinase (PI3K)/Akt pathway by hypoxia inducible factor-1 (HIF-1) resulting in the phosphorylation of eNOS at Ser-1177 along with the binding of calcium calmodulin and heat shock protein-90 (HSP-90) causing the electron to flow from reductase to oxygenase on eNOS resulting in the release of NO [77]. NO reacts with haem of soluble guanylyl cyclase which on activation relaxes smooth muscle cells [78]. Suppressed production or deficiency of nitric oxide inhibits the function of endothelial cells. eNOS deficiency has shown an elevated stromal-cell derived factor-1α (SDF-1α) and upregulation of CXCR-4 leading to the recruitment and proliferation of smooth muscle cells, which is an initial step in atherosclerotic plaque formation [79]. eNOS inhibition mechanism by proline rich tyrosine kinase 2 in response to fluid stress and insulin has also been reported [80]; Endostatin affects eNOS by dephosphorylating it at Ser-1177 residue inhibiting the endothelial cell migration [81] and endostatin is used for an anti-angiogenesis study in cancer [82].

**Fig. 1:**
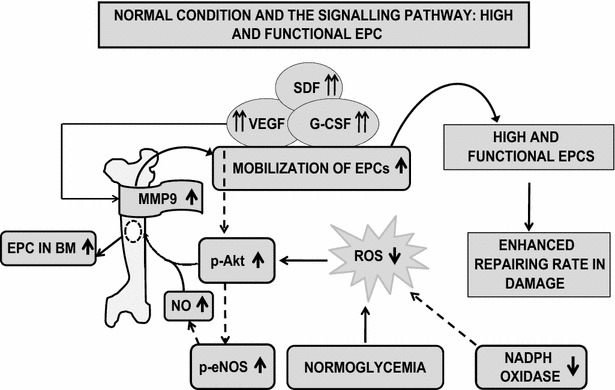
This schematic demonstrates healthy mobilization of EPC due to high cytokines, high p-Akt, high NO released by eNOS and low superoxide by NADPH oxidase

**Fig. 2:**
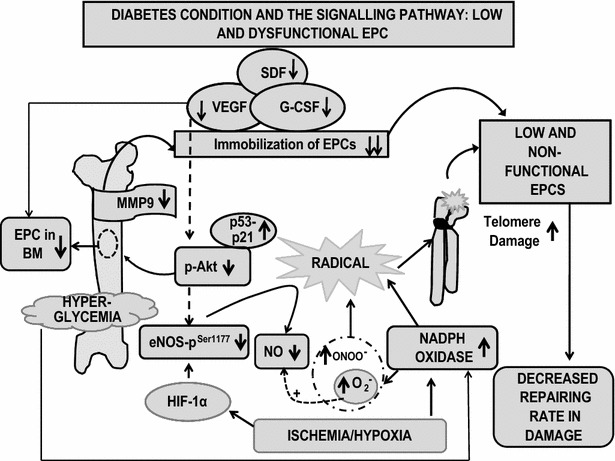
This schematic demonstrates defective mobilization of EPC and dysfunctional EPC due to high ROS (via eNOS and NADPH Oxidase) and high glucose in diabetes

There are several sources of ROS in blood vessels. Some of the sources are eNOS and NADPH Oxidase as shown in Figs. 1 and 2. These ROS contributes to damage the DNA which is called a telomere. Telomere shortening of endothelial progenitor cells (EPCs) may be the key factor in endothelial cell senescence. The rate of telomere shortening is highly dependent on cellular oxidative damage. Several reports suggest that EPC telomere shortening via increased oxidative DNA damage may play an important role in the pathogenesis of coronary artery disease. Therefore, it might be possible that telomere shortening via oxidative damage is the cause of the reduced number of EPC in diabetic patients.

## Other regulators of EPC in diabetes

Certain cytokines help in the reparative property of EPC like TNF and IL. In particular; reparative property of EPC is affected via IL-10 through modulation of miR-375 and CXCR4/SDF1 through HIF-2α [83]. Additionally, stromal cell derived factor 1(SDF 1) mediated EPC mobilization from bone marrow to damaged areas plays a critical role in angiogenesis [84]. Advanced glycation end product (AGEs) also may impair EPC migration and homing to damaged sites via syndecan 4 [85]. Syndecan 4 is a ubiquitous heparin sulfate proteoglycan on cell surface playing a critical role in inflammation. GTPCH I/BH4 pathway is critical to preserve EPC quantity, function, and regenerative capacity during wound healing [86]. Notch also plays a critical role in EPC function via locally generated TGFBIp at wound sites that may contribute to differentiation and angiogenic function of EPC via recruitment of EPC by regulating expression of DLL1 and JAG1 [87]. Neovascularization may also be facilitated by Notch through intracellular NAMPT-NAD(+)-SIRT1 cascade. Collectively, EPC function may be regulated by CXCR4/SDF1, AGE/Synd4, GTPCH and Notch.

Recently, microRNA(miR) have been reported to play a critical role in EPC function via silencing of gene expression. miR 10A and 21 modulate EPC senescence via HMGA2 [88]. miR107 inhibits EPC differentiation via HIF [89]. miR-126, 21, 27a, 27b and miR130 have been reported to be down regulated in EPC from DM patients. Anti miR-126 inhibited EPC proliferation, migration and enhanced apoptosis and restored miR-126 in EPC from diabetic patients resulted in proliferation, migration and EPC apoptotic ability [90]. miR-126 down regulation impairs EPC function via Spred-1 through Ras/ERK/VEGF and PI3K/Akt/eNOS signal pathway [91].

Altogether, TNF, IL, microRNA, signalling molecules like SDF/CXCR7, AGE/Syd4, Notch and GTCPH1 are important regulators of EPC function in diabetes. However, there are other regulators of EPC function like ROS level in diabetes.

## Effect of diabetes on ROS levels

Hyperglycemia has shown impaired function of circulating progenitor cell population [92, 93]. Although the precise mechanism still remains to be determined, there is an excessive production of reactive oxygen species (ROS) [94] due to the elevated vascular NADPH oxidase and eNOS uncoupling, which is the deviation in the eNOS reductase to oxygenase pathway resulting into the formation of superoxide instead of NO [95, 96] mediated by Protein Kinase C (PKC) pathway [97, 98] causing oxidative stress on cells. The uncoupling of eNOS results in impaired mobilization and function of EPCs [99, 100]. Increased asymmetric dimethylarginine (ADMA) reduces vasodilation by inhibiting NO production. High glucose induces the release of superoxide from mitochondria and NADPH oxidase via PKC activation where NO reacts with this superoxide resulting into the formation of peroxynitrite (ONOO^−^) which oxidizes a key eNOS cofactor called as tetrahydrobiopterin (BH_4_) [101, 102] causing decreased bioavailability of BH_4_ leading to uncoupling of eNOS [103, 104]. BH_4_ infusions restored endothelial dysfunction by enhancing NO production [105, 106]. Super oxide dismutase (SOD) present in mitochondria reacts with the superoxide resulting in the production of hydrogen peroxide (H_2_O_2_) [107]. The suppression of H_2_O_2_ by NO helps to suppress the superoxide produced by insulin stimulated NADPH activity by cGMP mediated mechanism making NO as antioxidant [108]. Advanced Glycation End Products when binds to the receptors (RAGE) causes diminished production of nitric oxide as it inhibits the NOS production causing endothelial dysfunction. This AGE-RAGE interaction is blocked by the soluble form of the receptor called sRAGE and low sRAGE indicates the progression of the diabetic atherosclerosis [109, 110].

Advanced glycation end products (AGE) oxidises low-density lipoproteins (LDL) to form oxidised LDL (oxLDL) E-selectin. One of the adhesion molecules from the selectin family is expressed by the activated endothelial cells which cause the ‘rolling’ of leukocytes on the endothelium. Monocyte chemoattractant protein-1 (MCP-1) recruits the monocytes mediated by nuclear factor kappa (NFκB) and the adhesion of these cells is taken care by vascular cell adhesion molecule-1 (VCAM-1). By means of ‘diapedesis’, these cells enter the vessel wall through the gaps between the endothelial cells where it converts into active macrophages and along with oxLDL form ‘foam’ cells, which become the trigger for atherosclerosis. The different sources of ROS and means of antioxidant generation contribute to the total ROS level in hyperglycemia condition.

## Telomere shortening in EPC

Telomere erosion is a key factor in endothelial cell senescence. Telomerase, a ribonucleoprotein is responsible for the attachment of telomeres (non-nucleosomal DNA protein) located at the terminal end of the chromosome and also helps in the addition of telomeric repeats (TTAGGG) every time a cell division occurs and serves as a protective cap with the help of RNA moiety [111, 112]. The telomere shortening occurs during the cell division as a result of semi-conservative DNA replication [113]. The three-dimensional T-Loop hides the termini from being identified as a broken double-stranded DNA, thus preventing the DNA repair mechanism getting activated [114, 115]. Absence of telomerase leads to the uncapping of telomere resulting in the structural change, up regulating cyclin dependent kinases p21CIP1 and p16INK4a activating p53 dependent response [116] and causing apoptosis [117]. Thus the telomerase hypothesis states that the shortening of the telomeres is the trigger for the mitotic clock for the cell senescence stopping the proliferation [118]. Human umbilical cord EPCs have shown 100 population doubling in vitro maintain intact telomerase activity [119]. Transfection of bone marrow derived endothelial cells with SV40T antigen; oncogenic N-Ras and hTERT showed uncontrollable cell proliferation leading to a transformation of endothelial cells to malignancy phenotype due to the upregulation of the telomerase activity and capping of the telomere ends [120].

Many study revealed that telomere shortening is directly associated with ageing and age dependent diseases like coronary artery disease and atherosclerosis [121–123]. Vascular dysfunction is associated with increased ICAM-1 and attenuated eNOS [124] and develops hypertension due to increase in endothelin-1 production [125] with reference to dysfunctional telomere and telomerase activity. This could be one of the primary causes in age related impaired angiogenesis [126], where the proliferative potential of the progenitor cells are decreased. Mice of telomerase deficient generations have shown shorter life span and impaired wound response [127, 128]. Kushner et al. [130] has shown EPCs from older adult humans have the low expression of PI3 Kinase/Akt, p70 S6-kinase and Bcl-2 and also 60% reduced telomerase activity [129].

Chronic oxidative stress pushes the telomere to lose its integrity leading to EPCs senescence and reduced number [130]. oxidised LDL induced EPCs/Ec where it undergoes premature cell death [131] via PI3 kinase/Akt pathway by regulating telomerase activity [132], and is a risk factor for the atherosclerotic lesions [133]. Angiotensin-2 causes EPCs senescence via gp91phox, which causes the increase in the oxidative stress due to the formation of peroxynitrite, eventually inactivating telomerase [134]. C-reactive protien (CRP) directly affects the stability of EPCs by acting on eNOS [135]. CRP also causes endothelial dysfunction by inhibiting the production of prostacyclin (potent vasodilator) [136] and increases the production of endothelin-1 and interleukin-6 [137]. But EPCs transfected with MnSOD-RNAi when undergone CRP treatment inhibited the production of ROS and thus stating that CRP induced ROS results in reduced TERT activity [138]. Cellular aging is caused due to the transport of telomerase reverse transcriptase (TERT) from nucleus to cytoplasm with the increase in the generation of superoxide. Some studies have shown improvising the telomerase and telomere function helps in the prevention of the cellular senescence associated with the EPCs [139, 140]. Scavenging of the superoxide anion helps to restore the telomere length by activating telomerase [141−143] IGF-1 which is a regulator of EPCs increases’ NOS by PI3 kinase/Akt signalling [144, 145]. Increase in the IGF-1 levels improvised the telomerase activity which opens a new window as a therapy for the disease related to dysfunctional progenitor cells [146]. Function and survival of EPCs can be improved by the high expression of Human TERT (hTERT) which could delay the cell senescence [140]. High-Density Lipoprotein (HDL) prevents the cell senescence by increasing the NO production and causing telomerase stability [147, 148]. Statins help in the phosphorylation of Akt, TERT, enhanced production of NOS, and activation of telomerase [149, 150]. Statins enhance the SIRT-1 expression via mitochondrial biogenesis by diminished production of mitochondrial ROS and H_2_O_2_ levels [151]. Also pioglitazone—drug prescribed for patients with type-2 diabetes [152], cardio-protective drug puerarin [153] and resveratrol [154] help in the reducing endothelial senescence by activation of telomerase and preventing endothelial apoptosis concluding that telomerase-telomere integrity is highly essential for the maintenance of cellular longevity and vascular homeostasis. Altogether maintaining telomere can be useful in maintain EPC number and function, hence drugs that can prevent telomere erosion can be useful in reducing endothelial cell senescence.

There are several drugs that have been reported to increase functional EPC via a number of other signaling pathways, which is discussed below.

## Novel and experimental drugs that increase EPC in diabetes condition

Treatment of diabetes by standard drug metformin, thiazolidinediones, DPP4, insulin, stain and ACE inhibitor may increase number and improve the function of EPC. The probable mechanism by which these drugs alter EPC function may involve the reduction in inflammation, oxidative stress, insulin resistance and NO bioavailability. Table 3 shows that vildagliptin, sitagliptin and aliskiren play a therapeutic role via cytokine SDF. On the other hand, amlodipine metformin, and simvastatin play a therapeutic role via eNOS while Pioglitazone plays a therapeutic role via ICAM and VCAM. Certain other therapeutic role can be initiated by cytokines and microRNA. Hence the low number and function of EPC can be improved by treatment of diabetes with available existing drugs and novel drugs.

**Table 3:** Drugs that increase EPC number and improve EPC function in diabetes condition

Drug	Signalling pathway targeted	Diabetes related disorder	References
Vildagliptin	SDF	Type 2 diabetes	Dei et al. [155] Cardiovasc Diabetol. 2017
Elevated CXCR7	Akt/GSK/Fyn	Limb ischemia	Dai et al.[156] Circ Res. 2017 [224]
Metformin	AMPK/NOS	Diabetes and wound healing	Yu et al. [157] Cardiovasc Diabetol. 2016
Amlodipine	VEGF/Akt/eNOS	Diabetes	Sun et al. [158] Biomed Res Int. 2016
Aliskiren	SDF	Diabetes	Chang et al. [159] PLoS One. 2015[227]
Insulin and Glargine	–	Diabetes	Oikonomou et al. [160] Cardiovasc Diabetol. 2014
Ginkgo Biloba extract	SOD	Diabetes	Zhao et al. [161]Genet Mol Res. 2014
Aliskiren	–	Diabetes	Raptis et al. [162] Am J Hypertens. 2015
Cathepsin B	GSK 3 beta	Diabetes	Hibbert et al. [163] Diabetes. 2014
Simvastatin	eNOS	Retinopathy	Zhang et al. [164] Exp Eye Res. 2012
Insulin		Diabetes	Dong et al. [165] Microvasc Res. 2011
Sitagliptin	SDF	Diabetes	Fadini et al. [166] Diabetes Care. 2010
Adiponectin	p38 MAPK/P16INK	Diabetes	Chang et al.[167] Diabetes. 2010
Pioglitazone	ICAM-1/VCAM-1	CV risk in Diabetes	Wang et al. [168] Am Heart J. 2006
Vitamin D	–	Diabetes	Yiu et al. [169] Atherosclerosis
Acarbose	Akt/eNOS	Diabetes and wound healing	Han et al. [170] Oxid Med Cell Longev
Crocetin	PI3K/Akt/ eNOS and ROS	Diabetes	Cao et al. [171] Life Sci

In DM patients in vitro, vitamin D supplementation improves EPC viability while oral supplementation of vitamin D significantly affects vascular function and contributed to functional EPC [169]. Apart from vitamin D, Acarbose which is a well-known oral glucose lowering drug displayed improved wound healing and angiogenic potential in DM mice via Akt/eNOS pathway [170]. In line with this observation for vitamin D and acarbose, crocetin enhances NO bioavailability via PI3K/Akt-eNOS and ROS pathway [171].

**Fig. 3:**
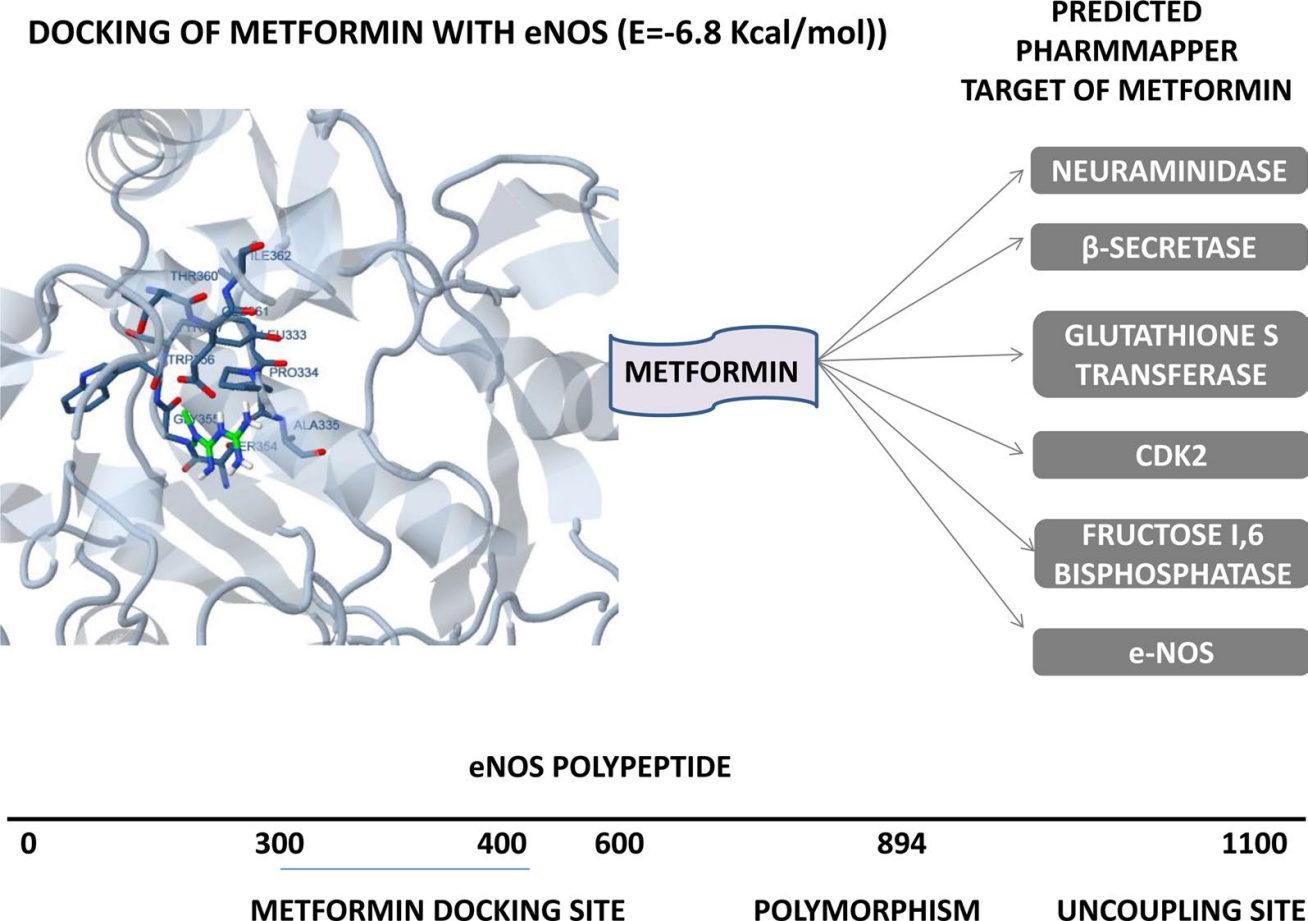
eNOS docking site (333, 334, 335,354, 355, 356, 357, 360, 361, 362) and polymorphism found in diabetes and uncoupling site

Several other drugs have been reported to increase the number and function of EPC in diabetes cases. Few have been listed in Table 3 such as vildagliptin, metformin, amlodipine, aliskiren, insulin and glargine, cathepsin, simvastatin, sitagliptin, adiponectin, pioglitazone. The drug vildagliptin, aliskiren, sitagliptin increases the level of EPC via increasing cytokine SDF [172] for the release of EPC. The other well known drug metformin works via AMPK/eNOS pathway while amlodipine works via VEGF/Akt/eNOS pathway. Simvastatin also works via eNOS pathway. On the other hand, Adiponectin acts via p38MAPK/p16INK pathway and Pioglitazone works via ICAM/VCAM pathway. All these drugs’ acts in such a way that EPC number and function is increased to repair the damage in diabetes condition. Metformin is one of the most popular medicines used in diabetic condition, and its targets’ protein has been shown in Fig. 3 via PharmMapper analysis such as eNOS, CDK2, neuraminidase, GST along with the protein mapping of eNOS for its binding site. All the target protein of metformin obtained via PharmMapper analysis, and its docking value with metformin have been shown in Table 4 and few have been shown in Fig. 3. Table 5 also lists that these proteins also plays a critical role in diabetes as polymorphism of these proteins have been found diabetic cases, and also inhibitors have been used to demonstrate significant results. Altogether, we can summarize that uncoupled eNOS leading to ROS generation both by PharmMapper analysis as well as Pubmed analysis. These proteins for release of EPC seem to be the common target affected by most of the drugs.

**Table 4:** Predicted target of metformin via PharmMapper and its docking value with different targets

Metformin								
S. no.	Protein name	EST. free energy of binding (kcal/mol)	EST. inhibition constant, Ki (mM)	vdW+ Hbond+ desolv energy (kcal/mol)	Electrostatic energy (kcal/mol)	Total intermol. energy (kcal/mol)	Frequency (%)	Interact. surface
1.	Beta secretase	−5.54	0.086	−2.56	−2.98	−5.54	80	346.622
2.	HSP 90	−4.09	1.01	−3.58	−0.50	−4.09	30	365.345
3.	Fructose-1,6-bisphosphatase	−2.93	7.15	−1.54	−1.39	−2.93	20	219.69
4.	Neuraminidase	+0.03	–	+0.00	+0.03	+0.03	30	17,111.543
5.	CDK 2	−3.28	3.97	−1.42	−1.86	−3.28	100	269.215
6.	Glutathione S transferase	−0.03	944.98	+0.00	−0.03	−0.03	40	10,475.752
7	eNOS	*−6.8*	*0.01*	*−3.68*	*−3.13*	*−6.82*	100	419.151

**Table 5:** Proteins affected in diabetes

Protein affected in diabetes and predicted by PharmMapper	Polymorphism/inhibitors	Function in diabetes	References
eNOS	T786C G894T	Uncoupling leads to ROS generation	Konsola et al. [173] Int J Cardiol. 2016
Glutathione S transferase	Ile 105Val	Reduces ROS	Mergani et al. [174] Biochem Genet. 2016
Fructose1,6 bisphosphatase	Inhibitor	Gluconeogenesis	Van et al. [175] Handb Exp Pharmacol. 2011
HSp90	Inhibitors	Therapeutic/protective effect for diabetes mediated atherosclerosis	Vazquez et al. [176] Clin Investig Arterioscler. 2017
VEGF	Gene polymorphism	Angiogenesis	Gonzalez et al. [177] Int J Ophthalmol. 2017
			Lai et al. [178] Sci Rep. 2017
SDF	Genetic	Cytokine in diabetic foot ulcer	Gene. 2015
Neuraminidase	Novel gene	Positive regulation for insulin signalling	Dridi et al. [179] Diabetes 2013
CDK2	Loss	Beta cell depletion	Kim et al. [180] J Biol Chem. 2017

## Targeting of EPC for the therapy of diabetes and diabetes-related disease

**Fig. 4:**
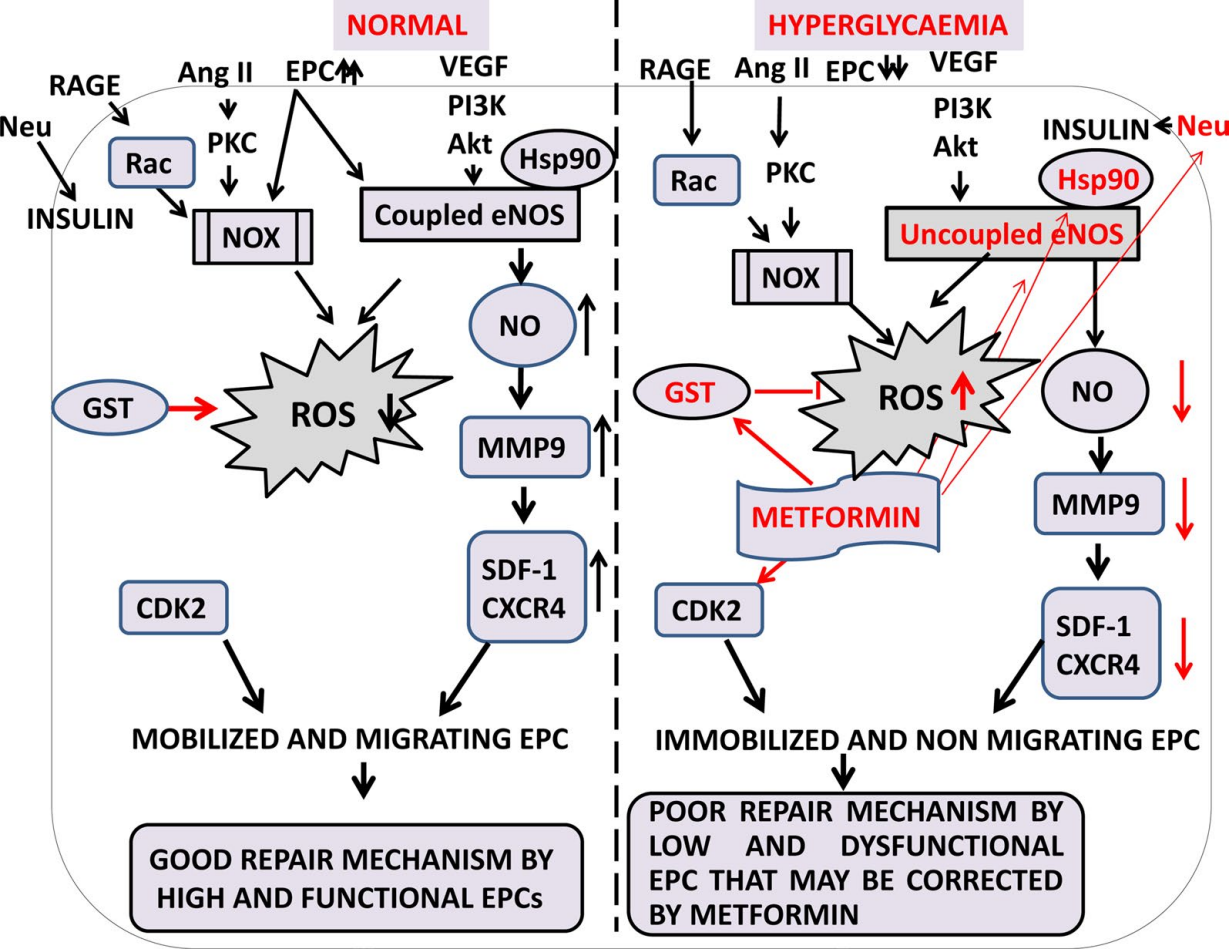
Signalling pathway in normal and hyperglycemia patients. Diabetes can be corrected by metformin. The major target of metformin is highlighted in *red* i.e. Uncoupled eNOS, HSp90, GST, CDK2, neuraminidase

The EPCs are targeted in diabetes via uncoupled eNOS and NADPH Oxidase(Nox) for radical oxygen generation(ROS) as shown in Fig. 4. eNOS level is controlled by AMPK in diabetes. This level of ROS is reduced by the glutathione S transferase (GST) level found in diabetic patients. Nox is regulated by PKC and Rac while eNOS is regulated by Akt and Hsp90 levels. Neuraminidase (Neu) positively regulates the insulin pathway. Metformin, a common drug used in diabetes targets whereas all of them together by targeting eNOS, GST, Hsp90, Neu-, beta secretase and Fructose 1,6 bisphosphatase, which explains the reason for the therapeutic value of metformin. The uncoupled eNOS leads to decrease NO availability and hence low level of MMP9, SDF-1 and immobilized and non-migrating EPC for the repair of diabetic related damage. The situation is reversed in normal patients where the high level of NO leads to high MMP9 and SDF-1 level and hence mobilized and functional EPC for repairing the damage caused in normal person.

The above signaling pathway explains the therapeutic value of metformin in mobilizing EPC in diabetes via eNOS-NO-MMP9-cytokine pathway to the damage site for therapy of damaged organ. Recently, reports also have demonstrated that metformin inhibits EPC migration by decreasing MMP 2 and 9 via MAPK/mTOR/autophagy pathway. Till the date, only one manuscript in the Pubmed demonstrates the negative effect of metformin in EPC migration while an uncountable number of publication report the positive role of metformin in diabetes and clinically metformin is still used for Diabetic patients. Hence in this regard, the role of metformin is slightly debatable regarding EPC migration and needs to be validated and confirmed in the future through enough experimental results. Due to scarcity of enough publication, the negative role of metformin needs to be validated in future and due to presence of enough publication for the positive role of metformin in diabetes and our own docking study for eNOS-Metformin, we advocate for the positive role of metformin in diabetes.

The HSp90 and eNOS forms complex in normal condition and in hyperglycemia, the complex disrupts. Hence, targeting Hsp90 and eNOS can be a good therapeutic site for treating diabetes. The uncoupled eNOS contributes to the total ROS pool and GST level reduces the total ROS level, hence GST can be a good therapeutic site for diabetes treatment. During normal condition, the cytokines are released to immobilize EPC from bone marrow; hence regulating cytokines can also have a remedial site for diabetes therapy. Altogether, for diabetes treatment, eNOS, Nox, HSp90, GST and cytokine be a therapeutic site for diabetes via different novel and experimental drugs.

## DM treatment improves the mobilization of EPC

DM treatment via vildagliptin, metformin, amlodipine, aliskiren, insulin and glargine, cathepsin, simvastatin, sitagliptin, adiponectin, pioglitazone, stain, insulin, ACE inhibitor improves the mobilization of EPC from the bone marrow by release of cytokine from the damaged site which attracts the EPC to the damaged site. It has been clearly shown in Table 3 and Figs. 1, 2 and 4 that the main mechanism of mobilization of EPC is via cytokines and PI3K/Akt/eNOS and ROS pathway. Hence it can be concluded that DM treatment via a wide range of drugs mainly focuses on the mobilization of EPC from BM [181] to the damaged site, increasing circulating EPC number and function for repair purpose at the damaged site.

## Conclusion

Therefore, we can conclude that high level of ROS leads to telomere damage. The high ROS level caused due to eNOS uncoupling, and NADPH Oxidase leads to low cell proliferation by Akt pathway. The low level of cytokine leads to low immobilization of EPC from bone marrow to circulating blood. Therefore, altogether diabetic EPCs are less in number and are dysfunctional, which explains the reason for defective repair system in diabetes and diabetes-related diseases like cardiovascular disease, hind limb ischemia, diabetic retinopathy and diabetic kidney failure. Our results demonstrate that commonly used drug metformin can convert the dysfunctional EPC to functional EPC via targeting the previously identified targets as well as few new target proteins.

## Abbreviations

eNOS: endothelial nitric oxide synthase
NOX: NADPH oxidase
EPC: endothelial progenitor cell
ROS: reactive oxygen species
HSP: heat shock protein
NO: nitric oxide

## References

[1] Asahara T, Murohara T, Sullivan A, Silver M, van der Zee R, Li T, Witzenbichler B, Schatteman G, Isner JM (1997). Isolation of putative progenitor endothelial cells for angiogenesis. Science.

[2] Pearson JD (2010). Endothelial progenitor cells—An evolving story. Microvasc Res.

[3] Galley HF, Webster NR (2004). Physiology of the endothelium. Br J Anaesth.

[4] Reyes M, Dudek A, Jahagirdar B, Koodie L, Marker PH, Verfaillie CM (2002). Origin of endothelial progenitors in human postnatal bone marrow. J Clin Invest.

[5] Mieno S, Clements RT, Boodhwani M, Sodha NR, Ramlawi B, Bianchi C, Sellke FW (2008). Characteristics and function of cryopreserved bone marrow derived endothelial progenitor cells. Ann Thorac Surg.

[6] Hess DC, Hill WD, Martin-Studdard A, Carroll J, Brailer J, Carothers J (2002). Bone marrow as a source of endothelial cells and NeuN-expressing cells after stroke. Stroke.

[7] Rustemeyer P, Wittkowski W, Greve B, Stehling M (2007). Flow-cytometric identification, enumeration, purification, and expansion of CD133^+^ and VEGF-R2^+^ endothelial progenitor cells from peripheral blood. J Immunoass Immunochem.

[8] Baudin B, Bruneel A, Bosselut N, Vaubourdolle M (2007). A protocol for isolation and culture of human umbilical vein endothelial cells. Nat Protoc.

[9] Marin V, Kaplanski G, Gres S, Farnarier C, Bongrand P (2001). Endothelial cell culture: protocol to obtain and cultivate human umbilical endothelial cells. J Immunol Methods.

[10] Eggermann J, Kliche S, Jarmy G, Hoffmann K, Mayr-Beyrlea U, Debatinb KM, Waltenbergera J, Beltingerb C (2003). Endothelial progenitor cell culture and differentiation in vitro: a methodological comparison using human umbilical cord blood. Cardiovasc Res.

[11] Ingram DA, Mead LE, Tanaka H, Meade V, Fenoglio A, Mortell K, Pollok K, Ferkowicz MJ, Gilley D, Yoder MC (2004). Identification of a novel hierarchy of endothelial progenitor cells using human peripheral and umbilical cord blood. Blood.

[12] Gehling UM, Ergün S, Schumacher U, Wagener C, Pantel K, Otte M, Schuch G, Schafhausen P, Mende T, Kilic N, Kluge K, Schafer B, Hossfeld DK, Fiedler W (2000). In vitro differentiation of endothelial cells from AC133 positive progenitor cells. Blood.

[13] Quirici N, Soligo D, Caneva L, Servida F, Bossolasco P, Deliliers GL (2001). Differentiation and expansion of endothelial cells from human bone marrow CD133^+^ cells. Br J Haematol.

[14] Timmermans F, Van Hauwermeiren F, De Smedt M, Raedt R, Plasschaert F, De Buyzere ML, Gillebert TC, Plum J, Vandekerckhove B (2007). Endothelial outgrowth cells are not derived from CD133^+^ cells or CD45^+^ hematopoietic precursors. Arterioscler Thromb Vasc Biol.

[15] Peichev M, Naiyer AJ, Pereira D, Zhu Z, Lane WJ, Williams M, Oz MC, Hicklin DJ, Witte L, Moore MA, Rafii S (2000). Expression of VEGFR-2 and AC133 by circulating human CD34^+^ cells identifies a population of functional endothelial precursors. Blood.

[16] Nikolova-Krstevski V, Bhasin M, Otu HH, Libermann T, Oettgen P (2008). Gene expression analysis of embryonic stem cells expressing VE-cadherin (CD144) during endothelial differentiation. BMC Genom.

[17] Loomans CJM, Wan H, de Crom R, van Haperen R, de Boer HC, Leenen PJM, Drexhage HA, Rabelink TJ, van Zonneveld AJ, Staal FJT (2006). Angiogenic murine endothelial progenitor cells are derived from a myeloid bone marrow fraction and can be identified by endothelial NO synthase expression. Arterioscler Thromb Vasc Biol.

[18] Voyta JC, Via DP, Butterfield CE, Zetter BR (1984). Identification and isolation of endothelial cells based on their increased uptake of acetylated-low density lipoprotein. J Cell Biol.

[19] Niwa K, Kado T, Sakai J, Karino T (2004). The effects of a shear flow on the uptake of LDL and acetylated LDL by an EC monoculture and an EC–SMC co-culture. Ann Biomed Eng.

[20] Nakul-Aquaronne D, Bayle J, Frelin C (2003). Coexpression of endothelial markers and CD14 by cytokine mobilized CD34^+^ cells under angiogenic stimulation. Cardiovasc Res.

[21] Untergasser G, Koeck R, Wolf D, Rumpold H, Ott H, Debbage P, Koppelstaetter C, Gunsilius E. CD34^+^/CD133^-^ circulating endothelial precursor cells (CEP): characterization, senescence and in vivo application. Exp Gerontol. 2006;41(6):600–8. 10.1016/j.exger.2006.03.019 16698211

[22] Capiod JC, Tournois C, Vitry F, Sevestre MA, Daliphard S, Reix T, Nguyen P, Lefrère JJ, Pignon B.Characterization and comparison of bone marrow and peripheral blood mononuclear cells used for cellular therapy in critical leg ischaemia: towards a new cellular product.;Vox Sang. 2009;96(3):256–65. 10.1111/j.1423-0410.2008.01138.x 19207166

[23] Hristov M, Erl W, Linder S, Weber PC. Endothelial progenitor cells: characterization, pathophysiology, and possible clinical relevance. J Cell Mol Med. 2004;8(4):498–8. 10.1111/j.1582-4934.2004.tb00474.x PMC6740289 15601578

[24] Lin Y, Weisdorf DJ, Solovey A, Hebbel RP; Origins of circulating endothelial cells and endothelial outgrowth from blood. J Clin Invest. 2000;105(1):71–7. 10.1172/JCI8071 PMC382587 10619863

[25] Ito C, Kumagai M, Manabe A, Coustan-Smith E, Raimondi SC, Behm FG, Murti KG, Rubnitz JE, Pui CH, Campana D.Hyperdiploid acute lymphoblastic leukemia with 51 to 65 chromosomes: a distinct biological entity with a marked propensity to undergo apoptosis. Blood. 1999;93(1):315–20. 9864176

[26] Khoo CP1, Valorani MG, Brittan M, Alison MR, Warnes G, Johansson U, Hawa M, Pozzilli P.; Characterization of endothelial progenitor cells in the NOD mouse as a source for cell therapies. Diabetes Metab Res Rev. 2009;25(1):89–3. doi:10.1002/dmrr.898. 10.1002/dmrr.898 19065604

[27] Hristov M, Erl W, Weber PC. Endothelial progenitor cells: isolation and characterization.;Trends Cardiovasc Med. 2003;13(5):201–6. 10.1016/s1050-1738(03)00077-x 12837583

[28] Guo H, Qiao Z, Su L, Zhu L, Wang H, Ma L.;Analysis of immune reconstitution in adults undergoing non-myeloablative allogeneic peripheral blood stem cell transplantation. Haematol. 2003;88(7):833–5. 12857567

[29] Yoon YS, Wecker A, Heyd L, Park JS, Tkebuchava T, Kusano K, Hanley A, Scadova H, Qin G, Cha DH, Johnson KL, Aikawa R, Asahara T, Losordo DW. Clonally expanded novel multipotent stem cells from human bone marrow regenerate myocardium after myocardial infarction. J Clin Invest. 2005;115(2):326–38. 10.1172/JCI22326 PMC546424 15690083

[30] Vittet D, Prandini MH, Berthier R, Schweitzer A, Martin-Sisteron H, Uzan G, Dejana E. Embryonic stem cells differentiate in vitro to endothelial cells through successive maturation steps. Blood. 1996;88(9):3424–31. 8896407

[31] Doetschman T, Shull M, Kier A, Coffin JD.;Embryonic stem cell model systems for vascular morphogenesis and cardiac disorders. Hypertens. 1993;22(4):618–29. 10.1161/01.hyp.22.4.618 8406668

[32] Li Z, Han Z, Wu JC. Transplantation of human embryonic stem cell-derived endothelial cells for vascular diseases. J Cell Biochem. 2009;106(2):194–9. 10.1002/jcb.22003 PMC2866109 19097085

[33] McCloskey KE, Smith DA, Jo H, Nerem RM. Embryonic stem cell-derived endothelial cells may lack complete functional maturation in vitro. J Vasc Res. 2006;43(5):411–21. 10.1159/000094791 16877873

[34] Glaser R, Peacock WF, Wu AH, Muller R, Möckel M, Apple FS.;Placental growth factor and B-type natriuretic peptide as independent predictors of risk from a multibiomarker panel in suspected acute coronary syndrome (Acute Risk and Related Outcomes Assessed With Cardiac Biomarkers [ARROW]) study. Am J Cardiol. 2011;107(6):821–6. 10.1016/j.amjcard.2010.11.003 21247525

[35] Schmidt D, Breymann C, Weber A, Guenter CI, Neuenschwander S, Zund G, Turina M, Hoerstrup SP. Umbilical cord blood derived endothelial progenitor cells for tissue engineering of vascular grafts. Ann Thorac Surg. 2004;78(6):2094–8. 10.1016/j.athoracsur.2004.06.052 15561042

[36] Murohara T, Ikeda H, Duan J, Shintani S, Sasaki Ki, Eguchi H, Onitsuka I, Matsui K, Imaizumi T. Transplanted cord blood-derived endothelial precursor cells augment postnatal neovascularization.; J Clin Invest. 2000;105(11):1527–36. 10.1172/JCI8296 PMC300847 10841511

[37] Ahrens I, Domeij H, Topcic D, Haviv I, Merivirta RM, Agrotis A, Leitner E, Jowett JB, Bode C, Lappas M, Peter K.; Successful in vitro expansion and differentiation of cord blood derived CD34+ cells into early endothelial progenitor cells reveals highly differential gene expression. PLoS One. 2011;6(8):e23210. 10.1371/journal.pone.0023210 PMC3155543 21858032

[38] Lin RZ, Dreyzin A, Aamodt K, Dudley AC, Melero-Martin JM. Functional endothelial progenitor cells from cryopreserved umbilical cord blood. Cell Transplant. 2011;20(4):515–22. 10.3727/096368910X532729 PMC3036780 20887663

[39] Jang JH, Kim SK, Choi JE, Kim YJ, Lee HW, Kang SY, Park JS, Choi JH, Lim HY, Kim HC.;Endothelial progenitor cell differentiation using cryopreserved, umbilical cord blood-derived mononuclear cells. Acta Pharmacol Sin. 2007;28(3):367–74. 10.1111/j.1745-7254.2007.00519.x 17302999

[40] Hughes SE. Functional characterization of the spontaneously transformed human umbilical vein endothelial cell line ECV304: use in an in vitro model of angiogenesis. Exp Cell Res. 1996;225(1):171–85. 10.1006/excr.1996.0168 8635510

[41] Bagley RG, Walter-Yohrling J, Cao X, Weber W, Simons B, Cook BP, Chartrand SD, Wang C, Madden SL, Teicher BA.Endothelial precursor cells as a model of tumor endothelium: characterization and comparison with mature endothelial cells.Cancer Res 2003;63(18):5866–73. 14522911

[42] Carvalho FA, Graça LM, Martins-Silva J, Saldanha C. Biochemical characterization of human umbilical vein endothelial cell membrane bound acetylcholinesterase. FEBS J. 2005;272(21):5584–94. 10.1111/j.1742-4658.2005.04953.x 16262697

[43] Hur J, Yoon CH, Kim HS, Choi JH, Kang HJ, Hwang KK, Oh BH, Lee MM, Park YB (2004). Characterization of two types of endothelial progenitor cells and their different contributions to neovasculogenesis. Arterioscler Thromb Vasc Biol.

[44] Mukai N, Akahori T, Komaki M, Li Q, Kanayasu-Toyoda T, Ishii-Watabe A, Kobayashi A, Yamaguchi T, Abe M, Amagasa T, Morita I (2008). A comparison of the tube forming potentials of early and late endothelial progenitor cells. Exp Cell Res.

[45] Yamamoto K, Takahashi T, Asahara T, Ohura N, Sokabe T, Kamiya A, Ando J (2003). Proliferation, differentiation, and tube formation by endothelial progenitor cells in response to shear stress. J Appl Physiol.

[46] Obi S, Yamamoto K, Shimizu N, Kumagaya S, Masumura T, Sokabe T, Asahara T, Ando J (2009). Fluid shear stress induces arterial differentiation of endothelial progenitor cells. J Appl Physiol.

[47] Kimiko Y, Sokabe T, Watabe T, Miyazono K, Yamashita JK, Obi S, Ohura N, Matsushita A, Kamiya A, Ando J (2005). Fluid shear stress induces differentiation of Flk-1-positive embryonic stem cells into vascular endothelial cells in vitro. Am J Physiol Heart Circ Physiol.

[48] Haga M, Chen A, Gortler D, Dardik A, Sumpio BE (2003). Shear stress and cyclic strain may suppress apoptosis in endothelial cells by different pathways. Endothelium.

[49] Fleming I, Fisslthaler B, Dixit M, Busse R (2005). Role of PECAM-1 in the shear-stress-induced activation of Akt and the endothelial nitric oxide synthase (eNOS) in endothelial cells. J Cell Sci.

[50] Aicher A, Heeschen C, Mildner-Rihm C, Urbich C, Ihling C, Technau-Ihling K, Zeiher AM, Dimmeler S (2003). Essential role of endothelial nitric oxide synthase for mobilization of stem and progenitor cells. Nat Med.

[51] Krubasik D, Eisenach PA, Kunz-Schughart LA, Murphy G, English WR (2008). Granulocyte-macrophage colony stimulating factor induces endothelial capillary formation through induction of membrane-type 1 matrix metalloproteinase expression in vitro. Int J Cancer.

[52] Powell TM, Paul JD, Hill JM, Thompson M, Benjamin M, Rodrigo M, McCoy JP, Read EJ, Khuu HM, Leitman SF, Finkel T, Cannon RO (2005). Granulocyte colony-stimulating factor mobilizes functional endothelial progenitor cells in patients with coronary artery disease. Arterioscler Thromb Vasc Biol.

[53] Wang QR, Wang F, Zhu WB, Lei J, Huang YH, Wang BH, Yan Q (2009). GM-CSF accelerates proliferation of endothelial progenitor cells from murine bone marrow mononuclear cells in vitro. Cytokine.

[54] Takahashi T, Kalka C, Masuda H, Chen D, Silver M, Kearney M, Magner M, Isner JM, Asahara T (1999). Ischemia and cytokine-induced mobilization of bone marrow-derived endothelial progenitor cells for neovascularization. Nat Med.

[55] Dimmeler S, Dernbach E, Zeiher A (2000). Phosphorylation of the endothelial nitric oxide synthase as Ser-1177 is Required for VEGF-induced endothelial cell migration. FEBS Lett.

[56] Asahara T, Takahashi T, Masuda H, Kalka C, Chen D, Iwaguro H, Inai Y, Silver M, Isner JM (1999). VEGF contributes to postnatal neovascularization by mobilizing bone marrow-derived endothelial progenitor cells. EMBO J.

[57] Heissig B, Hattori K, Dias S, Friedrich M, Ferris B, Hackett NR, Crystal RG, Besmer P, Lyden D, Moore MA, Werb Z, Rafii S (2002). Recruitment of stem and progenitor cells from the bone marrow niche requires MMP-9 mediated release of kit-ligand. Cell.

[58] Hojo Y, Ikeda U, Zhu Y, Okada M, Ueno S, Arakawa H, Fujikawa H, Katsuki TA, Shimada K (2000). Expression of vascular endothelial growth factor in patients with acute myocardial infarction. JACC.

[59] Ott I, Keller U, Knoedler M, Götze KS, Doss K, Fischer P, Urlbauer K, Debus G, von Bubnoff N, Rudelius M, Schömig A, Peschel C, Oostendorp RAJ (2005). Endothelial-like cells expanded from CD34+ blood cells improve left ventricular function after experimental myocardial infarction. FASEB J.

[60] Rookmaaker MB, Verhaar MC, Loomans CJ, Verloop R, Peters E, Westerweel PE, Murohara T, Staal FJ, van Zonneveld AJ, Koolwijk P, Rabelink TJ, van Hinsbergh VWM (2005). CD34^+^ cells home, proliferate, and participate in capillary formation, and in combination with CD34^−^ cells enhance tube formation in a 3-dimensional matrix. Arterioscler Thromb Vasc Biol.

[61] Grote K, Salguero G, Ballmaier M, Dangers M, Drexler H, Schieffer B (2007). The angiogenic factor CCN1 promotes adhesion and migration of circulating CD34^+^ progenitor cells: potential role in angiogenesis and endothelial regeneration. Blood.

[62] Oswald J, Boxberger S, Jørgensen B, Feldmann S, Ehninger G, Bornhäuser M, Wernera C (2004). Mesenchymal stem cells can be differentiated into endothelial cells in vitro. Stem Cells.

[63] Cacciatore F, Bruzzese G, Vitale DF, Liguori A, de Nigris F, Fiorito C, Infante T, Donatelli F, Minucci PB, Ignarro LJ, Napoli C (2011). Effects of ACE inhibition on circulating endothelial progenitor cells, vascular damage, and oxidative stress in hypertensive patients.;Eur. J Clin Pharmacol.

[64] Schmidt-Lucke C, Rössig L, Fichtlscherer S, Vasa M, Britten M, Kämper U, Dimmeler S, Zeiher AM (2005). Reduced number of circulating endothelial progenitor cells predicts future cardiovascular events: proof of concept for the clinical importance of endogenous vascular repair. Circulation.

[65] Vasa M, Fichtlscherer S, Aicher A, Adler K, Urbich C, Martin H, Zeiher A, Dimmeler S (2001). Number and migratory activity of circulating endothelial progenitor cells inversely correlate with risk factors for coronary artery disease. Circ Res.

[66] Hill JM, Zalos G, Halcox JP, Schenke WH, Waclawiw MA, Quyyumi AA, Finkel T (2003). Circulating endothelial progenitor cells, vascular function, and cardiovascular risk. N Engl J Med.

[67] Fadini GP, Miorin M, Facco M, Bonamico S, Baesso I, Grego F, Menegolo M, de Kreutzenberg SV, Tiengo A, Agostini C, Avogaro A (2005). Circulating endothelial progenitor cells are reduced in peripheral vascular complications of type 2 diabetes mellitus. J Am Coll Cardiol.

[68] Choi Jin-Ho, Kim KL, Huh W, Kim B, Byun J, Suh W, Sung J, Jeon ES, Oh HY, Kim DK (2004). Decreased number and impaired angiogenic function of endothelial progenitor cells in patients with chronic renal failure. Arterioscler Thromb Vasc Biol.

[69] Chen JZ, Zhang FR, Tao QM, Wang XX, Zhu JH, Zhu JH (2004). Number and activity of endothelial progenitor cells from peripheral blood in patients with hypercholesterolemia. Clin Sci.

[70] Matsumoto Y, Adams V, Walther C, Kleinecke C, Brugger P, Linke A, Walther T, Mohr FW, Schuler G (2009). Reduced number and function of endothelial progenitor cells in patients with aortic valve stenosis: a novel concept for valvular endothelial cell repair. Eur Heart J.

[71] Loomans CJ, van Haperen R, Duijs JM, Verseyden C, de Crom R, Leenen PJ, Drexhage HA, de Boer HC, de Koning KJ, Rabelink TJ, Staal FJ, van Zonneveld AJ (2009). Differentiation of bone marrow-derived endothelial progenitor cells is shifted into a proinflammatory phenotype by hyperglycemia. Mol Med.

[72] Suhara T, Mano T, Oliveira BE, Walsh K (2001). Phosphatidylinositol 3-Kinase/Akt signaling controls endothelial cell sensitivity to fas-mediated apoptosis via regulation of FLICE-inhibitory protein (FLIP). Circ Res.

[73] Lum H, Malik AB (1996). Mechanisms of increased endothelial permeability. Can J Physiol Pharmacol.

[74] Lampugnani MG, Dejana E (1997). Interendothelial junctions: structure, signalling and functional roles. Curr Opin Cell Biol.

[75] Ii M, Nishimura H, Iwakura A, Wecker A, Eaton E, Asahara T, Losordo DW (2005). Endothelial progenitor cells are rapidly recruited to myocardium and mediate protective effect of ischemic preconditioning via “imported” nitric oxide synthase activity. Circulation.

[76] Fitzgerald SM, Kemp-Harper BK, Parkington HC, Head GA, Evans RG (2007). Endothelial dysfunction and arterial pressure regulation during early diabetes in mice: roles for nitric oxide and endothelium derived hyperpolarizing factor. Am J Physiol Regul Integr Comp Physiol.

[77] Kimura M, Ueda K, Goto C, Jitsuiki D, Nishioka K, Umemura T, Noma K, Yoshizumi M, Chayama K, Higashi Y (2007). Repetition of ischemic preconditioning augments endothelium-dependent vasodilation in humans: role of endothelium-derived nitric oxide and endothelial progenitor cells. Arterioscler Thromb Vasc Biol.

[78] Albrecht EWJA, Stegeman CA, Heeringa P, Henning RH, van Goor H (2003). Protective role of endothelial nitric oxide synthase. J Pathol.

[79] Zhang LN, Wilson DW, da Cunha V, Sullivan ME, Vergona R, Rutledge JC, Wang YX (2006). Endothelial NO synthase deficiency promotes smooth muscle progenitor cells in association with upregulation of stromal cell-derived factor-1α in a mouse model of carotid artery ligation. Arterioscler Thromb Vasc Biol.

[80] Fisslthaler B, Loot AE, Mohamed A, Busse R, Fleming I (2008). Inhibition of endothelial nitric oxide synthase activity by proline-rich tyrosine kinase 2 in response to fluid shear stress and insulin. Circ Res.

[81] Urbich C, Reissner A, Chavakis E, Dernbach E, Haendeler J, Fleming I, Zeiher AM, Kaszkin M, Dimmeler S (2002). Dephosphorylation of endothelial nitric oxide synthase contributes to the anti-angiogenic effects of endostatin. FASEB J.

[82] O’Reilly MS, Boehm T, Shing Y, Fukai N, Vasios G, Lane WS, Flynn E, Birkhead JR, Olsen BR, Folkman J (1997). Endostatin: an endogenous inhibitor of angiogenesis and tumour growth. Cell.

[83] Garikipati VN, Krishnamurthy P, Verma SK, Khan M, Abramova T, Mackie AR, Qin G, Benedict C, Nickoloff E, Johnson J, Gao E, Losordo DW, Houser SR, Koch WJ, Kishore R (2015). Negative regulation of miR-375 by interleukin-10 enhances bone marrow-derived progenitor cell-mediated myocardial repair and function after myocardial infarction. Stem Cells.

[84] Cheng M, Huang K, Zhou J, Yan D, Tang YL, Zhao TC, Miller RJ, Kishore R, Losordo DW, Qin G (2015). A critical role of Src family kinase in SDF-1/CXCR4-mediated bone-marrow progenitor cell recruitment to the ischemic heart. J Mol Cell Cardiol.

[85] Xie J, Li R, Wu H, Chen J, Li G, Chen Q, Wei Z, He G, Wang L, Ferro A, Xu B (2017). Advanced glycation endproducts impair endothelial progenitor cell migration and homing via syndecan 4 shedding. Stem Cells.

[86] Tie L, Li XJ, Wang X, Channon KM, Chen AF (2009). Endothelium-specific GTP cyclohydrolase I overexpression accelerates refractory wound healing by suppressing oxidative stress in diabetes. Am J Physiol Endocrinol Metab.

[87] Sukmawati D, Tanaka R, Ito-Hirano R, Fujimura S, Hayashi A, Itoh S, Mizuno H, Daida H (2016). The role of Notch signaling in diabetic endothelial progenitor cells dysfunction. J Diabetes Complicat.

[88] Zhu S, Deng S, Ma Q, Zhang T, Jia C, Zhuo D, Yang F, Wei J, Wang L, Dykxhoorn DM, Hare JM, Goldschmidt-Clermont PJ, Dong C (2013). MicroRNA-10A* and MicroRNA-21 modulate endothelial progenitor cell senescence via suppressing high-mobility group A2. Circ Res.

[89] Meng S, Cao J, Wang L, Zhou Q, Li Y, Shen C, Zhang X, Wang C (2012). MicroRNA 107 partly inhibits endothelial progenitor cells differentiation via HIF-1β. PLoS ONE.

[90] Zhang T, Li L, Shang Q, Lv C, Wang C, Su B (2015). Circulating miR-126 is a potential biomarker to predict the onset of type 2 diabetes mellitus in susceptible individuals. Biochem Biophys Res Commun.

[91] Meng S, Cao JT, Zhang B, Zhou Q, Shen CX, Wang CQ (2012). Downregulation of microRNA-126 in endothelial progenitor cells from diabetes patients, impairs their functional properties, via target gene Spred-1. J Mol Cell Cardiol.

[92] Loomans CJM, de Koning EJ, Staal FJ, Rookmaaker MB, Verseyden C, de Boer HC, Verhaar MC, Braam B, Rabelink TJ, van Zonneveld AJ (2004). Endothelial progenitor cell dysfunction: a novel concept in the pathogenesis of vascular complications of type 1 diabetes. Diabetes.

[93] Kränkel N, Adams V, Linke A, Gielen S, Erbs S, Lenk K, Schuler G, Hambrecht R (2005). Hyperglycemia reduces survival and impairs function of circulating blood-derived progenitor cells. Arterioscler Thromb Vasc Biol.

[94] Cosentino F, Hishikawa K, Katusic ZS, Lüscher TF (1997). High glucose increases nitric oxide synthase expression and superoxide anion generation in human aortic endothelial cells. Circulation.

[95] Tomasz J, Mussa S, Gastaldi D, Sadowski J, Ratnatunga C, Pillai R, Channon KM (2002). Mechanisms of increased vascular superoxide production in human diabetes mellitus: role of NAD(P)H oxidase and endothelial nitric oxide synthase. Circulation.

[96] Pricci F, Leto G, Amadio L, Iacobini C, Cordone S, Catalano S, Zicari A, Sorcini M, Di Mario U, Pugliese G (2003). Oxidative stress in diabetes-induced endothelial dysfunction involvement of nitric oxide and protein kinase C. Free Radical Biol Med.

[97] Hirata K, Kuroda R, Sakoda T, Katayama M, Inoue N, Suematsu M, Kawashima S, Yokoyama M (1995). Inhibition of endothelial nitric oxide synthase activity by protein kinase C. Hypertension.

[98] Hink Ulrich, Li Huige, Mollnau Hanke, Oelze Mathias, Matheis Edi, Hartmann Mark, Skatchkov Mikhail, Thaiss Friedrich, Stahl RA, Warnholtz A, Meinertz T, Griendling K, Harrison DG, Forstermann U, Munzel T (2001). Mechanisms underlying endothelial dysfunction in diabetes mellitus. Circ Res.

[99] Thum T, Fraccarollo D, Schultheiss M, Froese S, Galuppo P, Widder JD, Tsikas D, Ertl G, Bauersachs J (2007). Endothelial nitric oxide synthase uncoupling impairs endothelial progenitor cell mobilization and function in diabetes. Diabetes.

[100] Chen YH, Lin SJ, Lin FY, Wu TC, Tsao CR, Huang PH, Liu PL, Chen YL, Chen JW (2007). High glucose impairs early and late endothelial progenitor cells by modifying nitric oxide-related but not oxidative stress-mediated mechanisms. Diabetes.

[101] Verhaar MC, Westerweel PE, van Zonneveld AJ, Rabelink TJ (2004). Free radical production by dysfunctional eNOS. Heart.

[102] Wever RMF, van Dam T, van Rijn HJM, de Groot F, Rabelink TJ (1997). Tetrahydrobiopterin regulates superoxide and nitric oxide generation by recombinant endothelial nitric oxide synthase. Biochem Biophys Res Commun.

[103] Kuzkaya N, Weissmann N, Harrison DG, Dikalov S (2003). Interactions of peroxynitrite, tetrahydrobiopterin, ascorbic acid, and thiols: implications for uncoupling endothelial nitric-oxide synthase. J Biol Chem.

[104] Landmesser U, Dikalov S, Price SR, McCann L, Fukai T, Holland SM, Mitch WE, Harrison DG (2003). Oxidation of tetrahydrobiopterin leads to uncoupling of endothelial cell nitric oxide synthase in hypertension. J Clin Invest.

[105] Alp NJ, Channon KM (2004). Regulation of endothelial nitric oxide synthase by tetrahydrobiopterin in vascular disease. Arterioscler Thromb Vasc Biol.

[106] Stroes E, Kastelein J, Cosentino F, Erkelens W, Wever R, Koomans H, Lüscher T, Rabelink T (1997). Tetrahydrobiopterin restores endothelial function in hypercholesterolemia. J Clin Invest.

[107] Cai S, Khoo J, Channon KM (2005). Augmented BH4 by gene transfer restores nitric oxide synthase function in hyperglycemic human endothelial cells. Cardiovasc Res.

[108] Bitar MS, Wahid S, Mustafa S, Al-Saleh E, Dhaunsi GS, Al-Mulla F (2005). Nitric oxide dynamics and endothelial dysfunction in type II model of genetic diabetes. Eur J Pharmacol.

[109] Zhuang D, Ceacareanu AC, Lin Y, Ceacareanu B, Dixit M, Chapman KE, Waters CM, Rao GN, Hassid A (2004). Nitric oxide attenuates insulin- or IGF-I-stimulated aortic smooth muscle cell motility by decreasing H2O2 levels: essential role of cGMP. Am J Physiol Heart Circ Physiol.

[110] Devangelio E, Santilli F, Formoso G (2007). Soluble RAGE in type 2 diabetes: association with oxidative stress. Free Radic Biol Med.

[111] Olivieri F, Mazzanti I, Abbatecola AM, Recchioni R, Marcheselli F, Procopio AD, Antonicelli R (2012). Telomere/telomerase system: a new target of statins pleiotropic effect?. Curr Vasc Pharmacol.

[112] Collins K (2000). Mammalian telomeres and telomerase. Curr Opin Cell Biol.

[113] Bodnar AG, Ouellette M, Frolkis M, Holt SE, Chiu CP, Morin GB, Harley CB, Shay JW, Lichtsteiner S, Wright WE (1998). Extension of life-span by introduction of telomerase into normal human cells. Science.

[114] Blasco MA (2005). Telomeres and human disease: ageing, cancer and beyond. Nat Rev Genet.

[115] Wong JMY, Collins K (2003). Telomere maintenance and disease. Lancet.

[116] Blasco MA (2007). Telomere length, stem cells and aging. Nat Chem Biol.

[117] Herbig U, Jobling WA, Chen BP, Chen DJ, Sedivy JM (2004). Telomere shortening triggers senescence of human cells through a pathway involving ATM, p53, and p21 (CIP1), but not p16(INK4a). Mol Cell.

[118] Rajaraman S, Choi J, Cheung P, Beaudry V, Moore H, Artandi SE (2007). Telomere uncapping in progenitor cells with critical telomere shortening is coupled to S-phase progression in vivo. Proc Natl Acad Sci.

[119] Campisi J, Kim SH, Lim CS, Rubio M (2001). Cellular senescence, cancer and aging: the telomere connection. Exp Gerontol.

[120] Ingram DA, Mead LE, Tanaka H, Meade V, Fenoglio A, Mortell K, Pollok K, Ferkowicz MJ, Gilley D, Yoder MC (2004). Identification of a novel hierarchy of endothelial progenitor cells using human peripheral and umbilical cord blood. Blood.

[121] MacKenzie KL, Franco S, Naiyer AJ, May C, Sadelain M, Rafii S, Moore MA (2002). Multiple stages of malignant transformation of human endothelial cells modelled by co-expression of telomerase reverse transcriptase, SV40 T antigen and oncogenic N-ras. Oncogene.

[122] Samani NJ, Boultby R, Butler R, Thompson JR, Goodall AH (2001). Telomere shortening in atherosclerosis. Lancet.

[123] Brouilette S, Singh RK, Thompson JR, Goodall AH, Samani NJ (2003). White cell telomere length and risk of premature myocardial infarction. Arterioscler Thromb Vasc Biol.

[124] van der Harst P, van der Steege G, de Boer RA, Voors AA, Hall AS, Mulder MJ, van Gilst WH, van Veldhuisen DJ, MERIT-HF Study Group (2007). Telomere length of circulating leukocytes is decreased in patients with chronic heart failure. J Am Coll Cardiol.

[125] Minamino T, Komuro I (2008). Role of telomeres in vascular senescence. Front Biosci.

[126] Pérez-Rivero G, Ruiz-Torres MP, Rivas-Elena JV, Jerkic M, Díez-Marques ML, Lopez-Novoa JM, Blasco MA, Rodríguez-Puyol D (2006). Mice deficient in telomerase activity develop hypertension because of an excess of endothelin production. Circulation.

[127] Franco S, Segura I, Riese HH, Blasco MA (2002). Decreased B16F10 melanoma growth and impaired vascularization in telomerase-deficient mice with critically short telomeres. Cancer Res.

[128] Blasco MA, Lee HW, Hande MP, Samper E, Lansdorp PM, DePinho RA, Greider CW (1997). Telomere shortening and tumor formation by mouse cells lacking telomerase RNA. Cell.

[129] Rudolph KL, Chang S, Lee HW, Blasco M, Gottlieb GJ, Greider C, DePinho RA (1999). Longevity, stress response, and cancer in aging telomerase-deficient mice. Cell.

[130] Kushner EJ, Maceneaney OJ, Weil BR, Greiner JJ, Stauffer BL, Desouza CA (2011). Aging is associated with a proapoptotic endothelial progenitor cell phenotype. J Vasc Res.

[131] Imanishi T, Hano T, Sawamura T, Nishio I (2004). Oxidized low-density lipoprotein induces endothelial progenitor cell senescence, leading to cellular dysfunction. Clin Exp Pharmacol Physiol.

[132] Kurz DJ, Decary S, Hong Y, Trivier E, Akhmedov A, Erusalimsky JD (2004). Chronic oxidative stress compromises telomere integrity and accelerates the onset of senescence in human endothelial cells. J Cell Sci.

[133] Breitschopf K, Zeiher AM, Dimmeler S (2001). Pro-atherogenic factors induce telomerase inactivation in endothelial cells through an Akt-dependent mechanism. FEBS Lett.

[134] Minamino T, Miyauchi H, Yoshida T, Ishida Y, Yoshida H, Komuro I (2002). Endothelial cell senescence in human atherosclerosis: role of telomere in endothelial dysfunction. Circulation.

[135] Imanishi T, Hano T, Nishio I (2005). Angiotensin II accelerates endothelial progenitor cell senescence through induction of oxidative stress. J Hypertens.

[136] Verma S, Kuliszewski MA, Li SH, Szmitko PE, Zucco L, Wang CH, Badiwala MV, Mickle DA, Weisel RD, Fedak PW, Stewart DJ, Kutryk MJ (2004). C-reactive protein attenuates endothelial progenitor cell survival, differentiation, and function: further evidence of a mechanistic link between C-reactive protein and cardiovascular disease. Circulation.

[137] Venugopal SK, Devaraj S, Jialal I (2003). C-reactive protein decreases prostacyclin release from human aortic endothelial cells. Circulation.

[138] Verma S, Li SH, Badiwala MV, Weisel RD, Fedak PW, Li RK, Dhillon B, Mickle DA (2002). Endothelin antagonism and interleukin-6 inhibition attenuate the proatherogenic effects of C-reactive protein. Circulation.

[139] Fujii H, Li SH, Szmitko PE, Fedak PW, Verma S (2006). C-reactive protein alters antioxidant defenses and promotes apoptosis in endothelial progenitor cells. Arterioscler Thromb Vasc Biol.

[140] Murasawa S, Llevadot J, Silver M, Isner JM, Losordo DW, Asahara T (2002). Constitutive human telomerase reverse transcriptase expression enhances regenerative properties of endothelial progenitor cells. Circulation.

[141] Furumoto K, Inoue E, Nagao N, Hiyama E, Miwa N (1998). Age-dependent telomere shortening is slowed down by enrichment of intracellular vitamin C via suppression of oxidative stress. Life Sci.

[142] Haendeler J, Hoffmann J, Diehl JF, Vasa M, Spyridopoulos I, Zeiher AM, Dimmeler S (2004). Antioxidants inhibit nuclear export of telomerase reverse transcriptase and delay replicative senescence of endothelial cells. Circ Res.

[143] Vasa M, Breitschopf K, Zeiher AM, Dimmeler S (2000). Nitric oxide activates telomerase and delays endothelial cell senescence. Circ Res.

[144] Withers DJ, Burks DJ, Towery HH, Altamuro SL, Flint CL, White MF (1999). Irs-2 coordinates Igf-1 receptor-mediated beta-cell development and peripheral insulin signalling. Nat Genet.

[145] Isenovic ER, Meng Y, Divald A, Milivojevic N, Sowers JR (2002). Role of phosphatidylinositol 3-kinase/Akt pathway in angiotensin II and insulin-like growth factor-1 modulation of nitric oxide synthase in vascular smooth muscle cells. Endocrine.

[146] Thum T, Hoeber S, Froese S, Klink I, Stichtenoth DO, Galuppo P, Jakob M, Tsikas D, Anker SD, Poole-Wilson PA, Borlak J, Ertl G, Bauersachs J (2007). Age-dependent impairment of endothelial progenitor cells is corrected by growth-hormone-mediated increase of insulin-like growth-factor-1. Circ Res.

[147] Noor R, Shuaib U, Wang CX, Todd K, Ghani U, Schwindt B, Shuaib A (2007). High-density lipoprotein cholesterol regulates endothelial progenitor cells by increasing eNOS and preventing apoptosis. Atherosclerosis.

[148] Pu DR, Liu L (2008). HDL slowing down endothelial progenitor cells senescence: a novel anti-atherogenic property of HDL. Med Hypotheses.

[149] Zhu JH, Chen JZ, Wang XX, Xie XD, Sun J, Zhang FR (2006). Homocysteine accelerates senescence and reduces proliferation of endothelial progenitor cells. J Mol Cell Cardiol.

[150] Assmus B, Urbich C, Aicher A, Hofmann WK, Haendeler J, Rössig L, Spyridopoulos I, Zeiher AM, Dimmeler S (2003). HMG-CoA reductase inhibitors reduce senescence and increase proliferation of endothelial progenitor cells via regulation of cell cycle regulatory genes. Circ Res.

[151] Ota H, Eto M, Kano MR, Kahyo T, Setou M, Ogawa S, Iijima K, Akishita M, Ouchi Y (2010). Induction of endothelial nitric oxide synthase, SIRT1, and catalase by statins inhibits endothelial senescence through the Akt pathway. Arterioscler Thromb Vasc Biol.

[152] Werner C, Gensch C, Pöss J, Haendeler J, Böhm M, Laufs U (2011). Pioglitazone activates aortic telomerase and prevents stress-induced endothelial apoptosis. Atherosclerosis.

[153] Zhu J, Wang X, Shang Y, Xie X, Zhang F, Chen J, Fu G (2008). Puerarin reduces endothelial progenitor cells senescence through augmentation of telomerase activity. Vascul Pharmacol.

[154] Xia L, Wang XX, Hu XS, Guo XG, Shang YP, Chen HJ, Zeng CL, Zhang FR, Chen JZ (2008). Resveratrol reduces endothelial progenitor cells senescence through augmentation of telomerase activity by Akt-dependent mechanisms. Br J Pharmacol.

[155] Dei Cas A, Spigoni V, Cito M, Aldigeri R, Ridolfi V, Marchesi E, Marina M, Derlindati E, Aloe R, Bonadonna RC, Zavaroni I. Vildagliptin, but not glibenclamide, increases circulating endothelial progenitor cell number: a 12-month randomized controlled trial in patients with type 2 diabetes. Cardiovasc Diabetol. 2017;16(1):27. 10.1186/s12933-017-0503-0 PMC5324295 28231835

[156] Dai X, Yan X, Zeng J, Chen J, Wang Y, Chen J, Li Y, Barati MT, Wintergerst KA, Pan K, Nystoriak MA, Conklin DJ, Rokosh G, Epstein PN, Li X, Tan Y. Elevating CXCR7 Improves Angiogenic Function of EPCs via Akt/GSK-3β/Fyn-Mediated Nrf2 Activation in Diabetic Limb Ischemia. Circ Res. 2017;120(5):e7–e23 10.1161/CIRCRESAHA.117.310619 PMC5336396 28137917

[157] Yu JW, Deng YP, Han X, Ren GF, Cai J, Jiang GJ. Metformin improves the angiogenic functions of endothelial progenitor cells via activating AMPK/eNOS pathway in diabetic mice. Cardiovasc Diabetol. 2016;15:88. 10.1186/s12933-016-0408-3 PMC4912824 27316923

[158] Sun J, Xie J, Kang L, Ferro A, Dong L, Xu B. Amlodipine Ameliorates Ischemia-Induced Neovascularization in Diabetic Rats through Endothelial Progenitor Cell Mobilization. Biomed Res Int. 2016;2016:3182764. 10.1155/2016/3182764 PMC4875975 27243031

[159] Chang TT, Wu TC, Huang PH, Lin CP, Chen JS, Lin LY, Lin SJ, Chen JW. Direct Renin Inhibition with Aliskiren Improves Ischemia-Induced Neovasculogenesis in Diabetic Animals via the SDF-1 Related Mechanism. PLoS One. 2015;10(8):e0136627. 10.1371/journal.pone.0136627 PMC4549314 26305217

[160] 28. Oikonomou D, Kopf S, von Bauer R, Djuric Z, Cebola R, Sander A, Englert S, Vittas S, Hidmark A, Morcos M, Korosoglou G, Nawroth PP, Humpert PM. Influence of insulin and glargine on outgrowth and number of circulating endothelial progenitor cells in type 2 diabetes patients: a partially double-blind, randomized, three-arm unicenter study. Cardiovasc Diabetol. 2014;13:137. 10.1186/s12933-014-0137-4 PMC4195950 25300286

[161] Zhao M, Wang XX, Wan WH. Effects of the ginkgo biloba extract on the superoxide dismutase activity and apoptosis of endothelial progenitor cells from diabetic peripheral blood. Genet Mol Res. 2014;13(1):220–7. 10.4238/2014.January.14.1 24446314

[162] Raptis AE, Markakis KP, Mazioti MC, Ikonomidis I, Maratou EP, Vlahakos DV, Kotsifaki EE, Voumvourakis AN, Tsirogianni AG, Lambadiari VA, Lekakis JP, Raptis SA, Dimitriadis GD. Effect of aliskiren on circulating endothelial progenitor cells and vascular function in patients with type 2 diabetes and essential hypertension. Am J Hypertens. 2015;28(1):22–9. 10.1093/ajh/hpu119 24994608

[163] Hibbert B, Lavoie JR, Ma X, Seibert T, Raizman JE, Simard T, Chen YX, Stewart D, O'Brien ER. Glycogen synthase kinase-3β inhibition augments diabetic endothelial progenitor cell abundance and functionality via cathepsin B: a novel therapeutic opportunity for arterial repair. Diabetes. 2014;63(4):1410–21. 10.2337/db13-0941 24296714

[164] Zhang W, Han Q, Chen S, Yan H. Effect of simvastatin on the amount and function of endothelial progenitor cells from bone marrow in diabetic rats. Zhonghua Yan Ke Za Zhi. 2012;48(11):1015–20. 23302277

[165] Dong L, Kang L, Ding L, Chen Q, Bai J, Gu R, Li L, Xu B. Insulin modulates ischemia-induced endothelial progenitor cell mobilization and neovascularization in diabetic mice.;Microvasc Res. 2011;82(3):227–36 10.1016/j.mvr.2011.09.006 21964072

[166] Fadini GP, Boscaro E, Albiero M, Menegazzo L, Frison V, de Kreutzenberg S, Agostini C, Tiengo A, Avogaro A. The oral dipeptidyl peptidase-4 inhibitor sitagliptin increases circulating endothelial progenitor cells in patients with type 2 diabetes: possible role of stromal-derived factor-1alpha. Diabetes Care. 2010;33(7):1607–9. 10.2337/dc10-0187 PMC2890368 20357375

[167] 35. Chang J, Li Y, Huang Y, Lam KS, Hoo RL, Wong WT, Cheng KK, Wang Y, Vanhoutte PM, Xu A. Adiponectin prevents diabetic premature senescence of endothelial progenitor cells and promotes endothelial repair by suppressing the p38 MAP kinase/p16INK4A signaling pathway. Diabetes. 2010;59(11):2949–59. 10.2337/db10-0582 PMC2963556 20802255

[168] Wang CH, Ting MK, Verma S, Kuo LT, Yang NI, Hsieh IC, Wang SY, Hung A, Cherng WJ. Pioglitazone increases the numbers and improves the functional capacity of endothelial progenitor cells in patients with diabetes mellitus. Am Heart J. 2006;152(6):1051.e1–8. 10.1016/j.ahj.2006.07.029 17161050

[169] Yiu YF, Yiu KH, Siu CW, Chan YH, Li SW, Wong LY, Lee SW, Tam S, Wong EW, Lau CP, Cheung BM (2013). Tse HF.;Randomized controlled trial of vitamin D supplement on endothelial function in patients with type 2 diabetes. Atherosclerosis..

[170] Han X, Deng Y, Yu J, Sun Y, Ren G, Cai J, Zhu J, Jiang G (2017). Acarbose accelerates wound healing via Akt/eNOS Signaling in db/db Mice stem cell. Res Ther.

[171] Cao W, Cui J, Li S, Zhang D, Guo Y, Li Q, Luan Y, Liu X (2017). Crocetin restores diabetic endothelial progenitor cell dysfunction by enhancing NO bioavailability via regulation of PI3K/AKT-eNOS and ROS pathways. Life Sci.

[172] Dhamodharan U, Viswanathan V, Krishnamoorthy E, Rajaram R, Aravindhan V. Genetic association of IL-6, TNF-α and SDF-1 polymorphisms with serum cytokine levels in diabetic foot ulcer. Gene. 2015;565(1):62–7. 10.1016/j.gene.2015.03.063 25839939

[173] Konsola T, Siasos G, Antonopoulos AS,, Kollia C, Oikonomou E, Tentolouris N, Gouliopoulos N, Vogiatzi G, Papamikroulis GA, Kassi E, Tousoulis D. The impact of T786C and G894T polymorphisms of eNOS on vascular endothelial growth factor serum levels in type 2 diabetes patients. Int J Cardiol. 2016;222:155–6. 10.1016/j.ijcard.2016.07.238 27494728

[174] Mergani A, Mansour AA, Askar T, Zahran RN, Mustafa AM, Mohammed MA, Saleh OM. Glutathione S-Transferase Pi-Ile 105 Val Polymorphism and Susceptibility to T2DM in Population from Turabah Region of Saudi Arabia. Biochem Genet. 2016;54(4):544–51. 10.1007/s10528-016-9740-2 27368697

[175] Van Poelje PD, Potter SC, Erion MD. Fructose-1, 6-bisphosphatase inhibitors for reducing excessive endogenous glucose production in type 2 diabetes. Handb Exp Pharmacol. 2011;203:279–1. 10.1007/978-3-642-17214-4_12 21484576

[176] Vázquez-Carrera M. HSP90 inhibitors as a future therapeutic strategy in diabetes-driven atherosclerosis. Clin Investig Arterioscler. 2017;29(2):67–8. 10.1016/j.arteri.2017.02.001 28274330

[177] Gonzalez-Salinas R, Garcia-Gutierrez MC, Garcia-Aguirre G, Morales-Canton V, Velez-Montoya R, Soberon-Ventura VR, Gonzalez V, Lechuga R, Garcia-Solis P, Garcia-Gutierrez DG, Garcia-Solis MV, Saenz de Viteri M, Solis-S JC. Evaluation of VEGF gene polymorphisms and proliferative diabetic retinopathy in Mexican population. Int J Ophthalmol. 2017;10(1):135–9 10.18240/ijo.2017.01.22 PMC5225362 28149790

[178] Lai CC, Wei KC, Chen WY, Mar GY, Wang WH, Wu CS, Tseng CJ, Yang KC, Chen LW, Liu CP. Risk Factors For Radiation-Induced Skin Ulceration in Percutaneous Coronary Interventions of Chronic Total Occluded Lesions: A 2-Year Observational Study. Sci Rep. 2017;7(1):8408. 10.1038/s41598-017-08945-4 PMC5559628 28814768

[179] Dridi L, Seyrantepe V, Fougerat A, Pan X, Bonneil E, Thibault P, Moreau A, Mitchell GA, Heveker N, Cairo CW, Issad T, Hinek A. Pshezhetsky AV Positive regulation of insulin signaling by neuraminidase 1. Diabetes. 2013;62(7):2338–46. 10.2337/db12-1825 PMC3712076 23520133

[180] Kim SY, Lee JH, Merrins MJ, Gavrilova O, Bisteau X, Kaldis P, Satin LS, Rane SG. Loss of Cyclin-dependent Kinase 2 in the Pancreas Links Primary β-Cell Dysfunction to Progressive Depletion of β-Cell Mass and Diabetes. J Biol Chem. 2017;292(9):3841–53. 10.1074/jbc.M116.754077 PMC5339765 28100774

[181] Asahara T, Masuda H, Takahashi T, Kalka C, Pastore C, Silver M, Kearne M, Magner M, Isner JM. Bone marrow origin of endothelial progenitor cells responsible for postnatal vasculogenesis in physiological and pathological neovascularization. Circ Res. 1999;85(3):221–8. 10.1161/01.res.85.3.221 10436164

